# 2-Amino­pyrimidinium hydrogen sulfate

**DOI:** 10.1107/S1600536811011123

**Published:** 2011-03-31

**Authors:** Adel Elboulali, Samah Toumi Akriche, Salem S. Al-Deyab, Mohamed Rzaigui

**Affiliations:** a Laboratoire de Chimie des Matériaux, Faculté des Sciences de Bizerte, 7021 Zarzouna Bizerte, Tunisia; b College of Science, King Saud University Riyadh, Saudi Arabia

## Abstract

In the crystal structure of the title compound, C_4_H_6_N_3_^+^·HSO_4_^−^, hydrogen sulfate anions self-assemble through O—H⋯O hydrogen bonds, forming chains along the *b* axis, while the cations form centrosymmetric pairs *via* N—H⋯N hydrogen bonds. The 2-amino­pyrimidinium pairs are linked to the sulfate anions *via* N—H⋯O hydrogen bonds, forming a two-dimensional network parallel to (10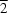). In addition, weak inter­molecular C—H⋯O contacts generate a three-dimensional network.

## Related literature

For the biological properties of pyrimidines, see: Rabie *et al.* (2007 ▶); Rival *et al.* (1991 ▶). For applications of amino­pyrimidines, see: Rospenk & Koll (2007 ▶). For amino­pyrimidine salts, see: Hemamalini *et al.* (2005 ▶); Childs *et al.* (2007 ▶); Lee *et al.* (2003 ▶); Ye *et al.* (2002 ▶). For sulfate salts with organic cations, see: Xu *et al.* (2009*a* ▶,*b* ▶).
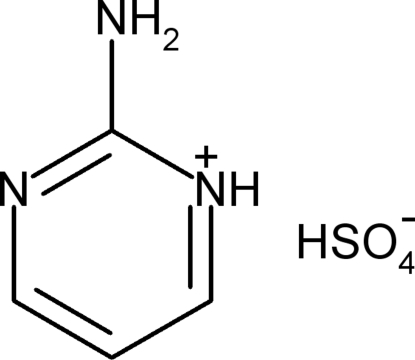


## Experimental

### 

#### Crystal data

C_4_H_6_N_3_^+^·HSO_4_^−^
*M* *_r_* = 193.19
Monoclinic, 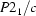

*a* = 8.388 (2) Å
*b* = 5.208 (3) Å
*c* = 18.468 (4) Å
β = 112.84 (2)°
*V* = 743.6 (5) Å^3^
*Z* = 4
Ag *K*α radiation
λ = 0.56087 Å
μ = 0.22 mm^−1^
*T* = 293 K
0.25 × 0.21 × 0.15 mm

#### Data collection

Enraf–Nonius CAD-4 diffractometer
3738 measured reflections
3647 independent reflections
2520 reflections with *I* > 2σ(*I*)
*R*_int_ = 0.015
2 standard reflections every 120 min  intensity decay: 1%

#### Refinement

*R*[*F* ^2^ > 2σ(*F* ^2^)] = 0.056
*wR*(*F* ^2^) = 0.159
*S* = 1.07
3647 reflections
110 parameters
H-atom parameters constrained
Δρ_max_ = 0.82 e Å^−3^
Δρ_min_ = −0.71 e Å^−3^

### 

Data collection: *CAD-4 EXPRESS* (Enraf–Nonius, 1994 ▶); cell refinement: *CAD-4 EXPRESS*; data reduction: *XCAD4* (Harms & Wocadlo, 1995) ▶; program(s) used to solve structure: *SHELXS86* (Sheldrick, 2008 ▶); program(s) used to refine structure: *SHELXL97* (Sheldrick, 2008 ▶); molecular graphics: *ORTEPIII* (Burnett & Johnson, 1996 ▶) and *DIAMOND* (Brandenburg & Putz, 2005 ▶); software used to prepare material for publication: *WinGX* publication routines (Farrugia, 1999 ▶).

## Supplementary Material

Crystal structure: contains datablocks I, global. DOI: 10.1107/S1600536811011123/lh5222sup1.cif

Structure factors: contains datablocks I. DOI: 10.1107/S1600536811011123/lh5222Isup2.hkl

Additional supplementary materials:  crystallographic information; 3D view; checkCIF report

## Figures and Tables

**Table 1:** Hydrogen-bond geometry (Å, °)

*D*—H⋯*A*	*D*—H	H⋯*A*	*D*⋯*A*	*D*—H⋯*A*
O1—H1⋯O4^i^	0.82	1.79	2.6100 (19)	174
N1—H1*B*⋯O1^ii^	0.86	2.38	3.140 (2)	148
N1—H1*B*⋯O4	0.86	2.58	3.155 (2)	125
N1—H1*A*⋯N3^iii^	0.86	2.16	3.017 (2)	172
N2—H2⋯O3	0.86	1.91	2.756 (2)	168
C2—H2*A*⋯O3^iv^	0.93	2.40	3.294 (2)	160
C3—H3⋯O2^v^	0.93	2.51	3.262 (3)	138
C4—H4⋯O4^vi^	0.93	2.53	3.316 (2)	142

Symmetry codes: (i) 
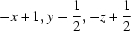
; (ii) 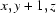; (iii) 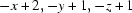; (iv) 
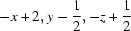
; (v) 
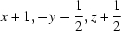
; (vi) 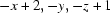.

**Fig. 1.**
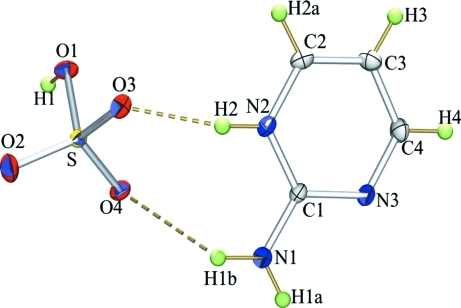
The asymmetric unit of (I). Displacement ellipsoids are drawn at the 30% probability level. H atoms are represented as spheres of arbitrary radii. Hydrogen bonds are represented as dashed lines.

**Fig. 2.**
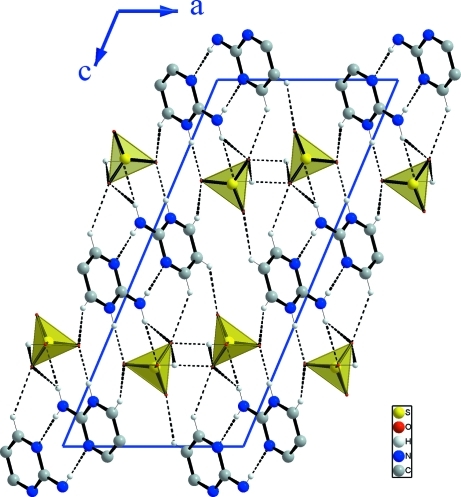
Projection of (I) along the b axis. The H-atoms not involved in H-bonding are omitted. H bonds are shown as dashed lines.

## supplementary crystallographic information

### Comment

Substantial attention has recently been focused on pyrimidine and its derivatives for their interesting properties as fungicides, vermicides, insecticides (Rabie *et al.*, 2007), antifungal agents and antiviral agents (Rival *et al.*, 1991). In particular, aminopyrimidines have been recognized as interesting nucleic bases, like cytosine, adenine and guanine which are responsible for molecular recognition and replication of DNA, through the formation and breakage of N—H···N hydrogen bonds (Rospenk & Koll, 2007). In continuation of our research on materials which could have interesting applications we report herein the synthesis and crystal structure of the title compound (I).

The asymmetric unit of the title compound (Fig. 1) consists of one hydrogen sulfate anion and one protonated 2-aminopyrimidine. The crystal packing of (I) is characterized by infinite chains built by HSO_4_^-^ anions extending along the b-direction. These chains are interconnected by cationic moieties *via* intermolecular N—H···O and C—H···O hydrogen bonds (Table 1) resulting in three-dimensional supra-molecular structure (Fig. 2).

As can be seen in table 1, the O1—H1···O4^i^ hydrogen bond links two adjacent hydrogen sulfate anions generating corrugated chains stacked along *c* axis (Fig. 2). In the sulfate anion, the S—O bond [1.569 (2) Å] involving the O atom bearing the acid H atom is longer than the other three S—O bonds, which range from 1.429 (1) to 1.459 (1) Å because of the bond multiplicity and the electronic mesomerism as reported previously in the hydrogen sulfate ion (Xu *et al.*, 2009*a*,b).

With regard to the organic framework, the neighbouring cations of 2-aminopyrimidine linked by the hydrogen bonds N1–H1A···N3 (2 - *x*, 1 - *y*, 1 - *z*) and N3···H1A–N1 (2 - *x*, 1 - *y*, 1 - *z*) form the cyclic dimer of [C_4_N_2_H_4_NH_2_]^2+^_2_. The cationic arrangement in crystal structure of 2-amino-4,6-dimethylpyrimidinium hydrogen sulfate (Hemamalini *et al.*, 2005) is closely related to that seen in the title compound. The dimers of the 2-aminopyrimidinium cations with planar rings (r.m.s. deviation = 0.008 Å) are connected to HSO_4_^-^ chains by hydrogen bonds N1–H1B···O4, N1–H1B···O1 (*x*, *y* + 1, *z*) and N2–H2···O3 to form a two-dimensional network (Fig. 2) which is linked into a three-dimensional network through weak intermolecular hydrogen bonds. These observations are similar to that of other 2-aminopyrimidinium salts (Childs *et al.*, 2007; Lee *et al.*, 2003; Ye *et al.*, 2002).

### Experimental

To a solution of 2-aminopyrimidine (0.19 g, 2 mmol) dissolved in a mixture of water/ethanol (10/5 ml) was added dropwise 2 mmol (0.15 ml) of commercial H_2_SO_4_ (98%, Aldrich). The reaction mixture was stirred and left under slowly evaporation at room temperature until formation of large colorless single crystals of the title compound.

### Refinement

All H atoms attached to C, N and O atoms were fixed geometrically and treated as riding with C—H = 0.93 Å, N—H= 0.86 Å and O—H = 0.82 Å with *U*_iso_(H) = 1.2 *U*_eq_(C, N) or 1.5 *U*_eq_(O)

### Crystal data

**Table:**

C_4_H_6_N_3_^+^·HSO_4_^−^	*F*(000) = 400
*M**_r_* = 193.19	*D*_x_ = 1.726 Mg m^−^^3^
Monoclinic, *P*2_1_/*c*	Ag *K*α radiation, λ = 0.56087 Å
Hall symbol: -P 2ybc	Cell parameters from 25 reflections
*a* = 8.388 (2) Å	θ = 9–11°
*b* = 5.208 (3) Å	µ = 0.22 mm^−^^1^
*c* = 18.468 (4) Å	*T* = 293 K
β = 112.84 (2)°	Prism, colorless
*V* = 743.6 (5) Å^3^	0.25 × 0.21 × 0.15 mm
*Z* = 4	

### Data collection

**Table:**

Enraf–Nonius CAD-4 diffractometer	*R*_int_ = 0.015
Radiation source: fine-focus sealed tube	θ_max_ = 28.0°, θ_min_ = 2.1°
graphite	*h* = −14→13
non–profiled ω scans	*k* = −8→0
3738 measured reflections	*l* = −30→13
3647 independent reflections	2 standard reflections every 120 min
2520 reflections with *I* > 2σ(*I*)	intensity decay: 1%

### Refinement

**Table:**

Refinement on *F*^2^	Primary atom site location: structure-invariant direct methods
Least-squares matrix: full	Secondary atom site location: difference Fourier map
*R*[*F*^2^ > 2σ(*F*^2^)] = 0.056	Hydrogen site location: inferred from neighbouring sites
*wR*(*F*^2^) = 0.159	H-atom parameters constrained
*S* = 1.07	*w* = 1/[σ^2^(*F*_o_^2^) + (0.0919*P*)^2^ + 0.0037*P*] where *P* = (*F*_o_^2^ + 2*F*_c_^2^)/3
3647 reflections	(Δ/σ)_max_ < 0.001
110 parameters	Δρ_max_ = 0.82 e Å^−^^3^
0 restraints	Δρ_min_ = −0.71 e Å^−^^3^

### Special details

**Table:**

Geometry. All e.s.d.'s (except the e.s.d. in the dihedral angle between two l.s. planes) are estimated using the full covariance matrix. The cell e.s.d.'s are taken into account individually in the estimation of e.s.d.'s in distances, angles and torsion angles; correlations between e.s.d.'s in cell parameters are only used when they are defined by crystal symmetry. An approximate (isotropic) treatment of cell e.s.d.'s is used for estimating e.s.d.'s involving l.s. planes.
Refinement. Refinement of *F*^2^ against ALL reflections. The weighted *R*-factor *wR* and goodness of fit *S* are based on *F*^2^, conventional *R*-factors *R* are based on *F*, with *F* set to zero for negative *F*^2^. The threshold expression of *F*^2^ > σ(*F*^2^) is used only for calculating *R*-factors(gt) *etc*. and is not relevant to the choice of reflections for refinement. *R*-factors based on *F*^2^ are statistically about twice as large as those based on *F*, and *R*- factors based on ALL data will be even larger.

### Fractional atomic coordinates and isotropic or equivalent isotropic displacement parameters (Å^2^)

**Table:**

	*x*	*y*	*z*	*U*_iso_*/*U*_eq_	
S	0.67465 (5)	−0.13645 (8)	0.21545 (2)	0.02768 (11)	
O1	0.66596 (17)	−0.4361 (3)	0.22175 (9)	0.0400 (3)	
H1	0.5717	−0.4774	0.2218	0.060*	
O2	0.5491 (2)	−0.0595 (3)	0.14085 (8)	0.0515 (4)	
O3	0.85241 (16)	−0.0898 (3)	0.22478 (7)	0.0365 (3)	
O4	0.64241 (17)	−0.0315 (3)	0.28174 (8)	0.0408 (3)	
N1	0.9071 (2)	0.3742 (3)	0.39016 (9)	0.0430 (4)	
H1A	0.8905	0.4952	0.4181	0.052*	
H1B	0.8390	0.3583	0.3416	0.052*	
N2	1.06403 (19)	0.0245 (3)	0.37781 (8)	0.0319 (3)	
H2	0.9966	0.0117	0.3291	0.038*	
N3	1.1419 (2)	0.2419 (3)	0.49787 (8)	0.0358 (3)	
C1	1.0366 (2)	0.2136 (3)	0.42160 (9)	0.0295 (3)	
C2	1.1941 (2)	−0.1444 (3)	0.40855 (11)	0.0376 (3)	
H2A	1.2105	−0.2737	0.3774	0.045*	
C3	1.3011 (3)	−0.1248 (4)	0.48528 (12)	0.0429 (4)	
H3	1.3912	−0.2404	0.5085	0.051*	
C4	1.2703 (3)	0.0753 (4)	0.52752 (10)	0.0418 (4)	
H4	1.3442	0.0939	0.5799	0.050*	

### Atomic displacement parameters (Å^2^)

**Table:**

	*U*^11^	*U*^22^	*U*^33^	*U*^12^	*U*^13^	*U*^23^
S	0.03166 (18)	0.02383 (17)	0.02574 (16)	−0.00177 (13)	0.00914 (13)	−0.00139 (13)
O1	0.0418 (7)	0.0245 (5)	0.0581 (8)	−0.0021 (5)	0.0240 (6)	−0.0020 (5)
O2	0.0539 (9)	0.0493 (9)	0.0337 (6)	−0.0035 (7)	−0.0023 (6)	0.0068 (6)
O3	0.0380 (6)	0.0388 (7)	0.0365 (6)	−0.0084 (5)	0.0186 (5)	−0.0057 (5)
O4	0.0423 (7)	0.0420 (7)	0.0409 (6)	0.0016 (5)	0.0192 (5)	−0.0115 (5)
N1	0.0456 (8)	0.0428 (9)	0.0325 (7)	0.0091 (7)	0.0062 (6)	−0.0061 (6)
N2	0.0408 (7)	0.0292 (6)	0.0263 (5)	−0.0040 (5)	0.0135 (5)	−0.0039 (5)
N3	0.0420 (7)	0.0361 (8)	0.0250 (6)	−0.0001 (6)	0.0084 (5)	−0.0043 (5)
C1	0.0361 (7)	0.0266 (6)	0.0255 (6)	−0.0042 (6)	0.0116 (5)	−0.0025 (5)
C2	0.0461 (9)	0.0287 (7)	0.0438 (9)	−0.0005 (7)	0.0238 (8)	−0.0030 (7)
C3	0.0450 (9)	0.0396 (10)	0.0436 (9)	0.0093 (8)	0.0168 (8)	0.0075 (8)
C4	0.0439 (9)	0.0468 (10)	0.0289 (7)	0.0016 (8)	0.0078 (7)	0.0022 (7)

### Geometric parameters (Å, °)

**Table:**

S—O2	1.4288 (14)	N2—C1	1.350 (2)
S—O3	1.4535 (13)	N2—H2	0.8600
S—O4	1.4588 (13)	N3—C4	1.324 (3)
S—O1	1.5690 (17)	N3—C1	1.349 (2)
O1—H1	0.8200	C2—C3	1.355 (3)
N1—C1	1.314 (2)	C2—H2A	0.9300
N1—H1A	0.8600	C3—C4	1.385 (3)
N1—H1B	0.8600	C3—H3	0.9300
N2—C2	1.343 (2)	C4—H4	0.9300
			
O2—S—O3	113.94 (9)	C4—N3—C1	117.25 (16)
O2—S—O4	113.37 (10)	N1—C1—N3	119.05 (16)
O3—S—O4	110.72 (8)	N1—C1—N2	120.26 (15)
O2—S—O1	108.21 (9)	N3—C1—N2	120.69 (16)
O3—S—O1	103.46 (8)	N2—C2—C3	119.50 (17)
O4—S—O1	106.34 (9)	N2—C2—H2A	120.3
S—O1—H1	109.5	C3—C2—H2A	120.3
C1—N1—H1A	120.0	C2—C3—C4	116.90 (18)
C1—N1—H1B	120.0	C2—C3—H3	121.5
H1A—N1—H1B	120.0	C4—C3—H3	121.5
C2—N2—C1	121.60 (15)	N3—C4—C3	124.04 (17)
C2—N2—H2	119.2	N3—C4—H4	118.0
C1—N2—H2	119.2	C3—C4—H4	118.0

### Hydrogen-bond geometry (Å, °)

**Table:**

*D*—H···*A*	*D*—H	H···*A*	*D*···*A*	*D*—H···*A*
O1—H1···O4^i^	0.82	1.79	2.6100 (19)	174
N1—H1B···O1^ii^	0.86	2.38	3.140 (2)	148
N1—H1B···O4	0.86	2.58	3.155 (2)	125
N1—H1A···N3^iii^	0.86	2.16	3.017 (2)	172
N2—H2···O3	0.86	1.91	2.756 (2)	168
C2—H2A···O3^iv^	0.93	2.40	3.294 (2)	160
C3—H3···O2^v^	0.93	2.51	3.262 (3)	138
C4—H4···O4^vi^	0.93	2.53	3.316 (2)	142

Symmetry codes: (i) −*x*+1, *y*−1/2, −*z*+1/2; (ii) *x*, *y*+1, *z*; (iii) −*x*+2, −*y*+1, −*z*+1; (iv) −*x*+2, *y*−1/2, −*z*+1/2; (v) *x*+1, −*y*−1/2, *z*+1/2; (vi) −*x*+2, −*y*, −*z*+1.

## References

[bb1] Brandenburg, K. & Putz, H. (2005). *DIAMOND*, Crystal impact GbR, Bonn, Germany.

[bb2] Burnett, M. N. & Johnson, C. K. (1996). *ORTEPIII* Report ORNL-6895. Oak Ridge National Laboratory, Tennessee, USA.

[bb3] Childs, S. L., Stahly, G. P. & Park, A. (2007). *Mol. Pharm.* **4**, 323–338. 10.1021/mp0601345 17461597

[bb4] Enraf–Nonius (1994). *CAD-4 EXPRESS* Enraf–Nonius, Delft, The Netherlands.

[bb5] Farrugia, L. J. (1999). *J. Appl. Cryst.* **32**, 837–838.

[bb6] Harms, K. & Wocadlo, S. (1995). *XCAD4* University of Marburg, Germany.

[bb7] Hemamalini, M., Mu­thiah, P. T., Rychlewska, U. & Plutecka, A. (2005). *Acta Cryst.* C**61**, o95–o97. 10.1107/S0108270104030975 15695921

[bb8] Lee, J.-H. P., Lewis, B. D., Mendes, J. M., Turnbull, M. M. & Awwadi, F. F. (2003). *J. Coord. Chem.* **56**, 1425–1442.

[bb9] Rabie, U. M., Abou-El-Wafa, M. H. & Mohamed, R. A. (2007). *J. Mol. Struct.* **871**, 6–13.

[bb10] Rival, Y., Grassy, G., Taudou, A. & Ecalle, R. (1991). *Eur. J. Med. Chem.* **26**, 13–18.

[bb11] Rospenk, M. & Koll, A. (2007). *J. Mol. Struct.* **844–845**, 232–241.

[bb12] Sheldrick, G. M. (2008). *Acta Cryst.* A**64**, 112–122. 10.1107/S0108767307043930 18156677

[bb13] Xu, Y.-M., Gao, S. & Ng, S. W. (2009*a*). *Acta Cryst.* E**65**, o3146. 10.1107/S1600536809048521 PMC2971874 21578865

[bb14] Xu, Y.-M., Gao, S. & Ng, S. W. (2009*b*). *Acta Cryst.* E**65**, o3147. 10.1107/S1600536809048545 PMC2972122 21578866

[bb15] Ye, M.-D., Hu, M.-L. & Ye, C.-P. (2002). *Z. Kristallogr. New Cryst. Struct.* **217**, 501–502.

